# Design, Development, and Evaluation of 5G-Enabled Vehicular Services: The 5G-HEART Perspective †

**DOI:** 10.3390/s22020426

**Published:** 2022-01-06

**Authors:** Grigorios Kakkavas, Maria Diamanti, Adamantia Stamou, Vasileios Karyotis, Faouzi Bouali, Jarno Pinola, Olli Apilo, Symeon Papavassiliou, Klaus Moessner

**Affiliations:** 1 School of Electrical and Computer Engineering, National Technical University of Athens, Iroon Polytechniou 9, 15780 Athens, Greece; gkakkavas@netmode.ntua.gr (G.K.); mdiamanti@netmode.ntua.gr (M.D.); stamouad@mail.ntua.gr (A.S.); 2 Department of Informatics, Ionian University, 49100 Corfu, Greece; karyotis@ionio.gr; 3 Institute for Future Transport & Cities, Coventry University, Coventry CV1 5FB, UK; ad6501@coventry.ac.uk; 4 VTT Technical Research Centre of Finland Ltd., Kaitoväylä 1, 90570 Oulu, Finland; jarno.pinola@vtt.fi (J.P.); olli.apilo@vtt.fi (O.A.); 5 Professorship for Communications Engineering, Technical University Chemnitz, Str. der Nationen 62, 09111 Chemnitz, Germany; klaus.moessner@etit.tu-chemnitz.de

**Keywords:** 5G mobile communications, network requirements, key performance indicators, transport vertical, network slicing, validation trials, vehicular services

## Abstract

The ongoing transition towards 5G technology expedites the emergence of a variety of mobile applications that pertain to different vertical industries. Delivering on the key commitment of 5G, these diverse service streams, along with their distinct requirements, should be facilitated under the same unified network infrastructure. Consequently, in order to unleash the benefits brought by 5G technology, a holistic approach towards the requirement analysis and the design, development, and evaluation of multiple concurrent vertical services should be followed. In this paper, we focus on the Transport vertical industry, and we study four novel vehicular service categories, each one consisting of one or more related specific scenarios, within the framework of the “5G Health, Aquaculture and Transport (5G-HEART)” 5G PPP ICT-19 (Phase 3) project. In contrast to the majority of the literature, we provide a holistic overview of the overall life-cycle management required for the realization of the examined vehicular use cases. This comprises the definition and analysis of the network Key Performance Indicators (KPIs) resulting from high-level user requirements and their interpretation in terms of the underlying network infrastructure tasked with meeting their conflicting or converging needs. Our approach is complemented by the experimental investigation of the real unified 5G pilot’s characteristics that enable the delivery of the considered vehicular services and the initial trialling results that verify the effectiveness and feasibility of the presented theoretical analysis.

## 1. Introduction

The deployment of the Fifth Generation (5G) cell sites, overlaid or underlaid with the conventional Fourth Generation (4G) cellular network, has already become a reality. This network evolution has motivated the emergence of a wide spectrum of diverse mobile applications, fostering the growth of several individual vertical industries [1]. As a consequence, 5G technology aims to not only provide extended connectivity with increased data rates and low latency but also to guarantee the seamless concurrent operation of these diverse applications over the same underlying network infrastructure. As a means of meeting the different requirements imposed by the different vertical industries and their applications, the conventional network architecture should shift from a single unpartitioned entity to several logical partitions (commonly referred to as network slices, each of which has its own virtual resources and targets the provisioning of customized network services to one or a combination of multiple verticals with overlapping prerequisites). In this context, a holistic approach targeting the requirement analysis, the architectural design and planning of the network infrastructure, as well as the effective trailing of the envisioned vertical industries’ services, considering multiple vertical sectors at the same time, is imperative.

Driven by the need for such holistic approaches, the “5G HEalth, AquacultuRe and Transport (5G-HEART) Validation Trials” Horizon 2020 project [2] aims to fill this research gap by defining and validating the 5G-converged network concepts that enable an intelligent hub of several supported vertical industries. In particular, 5G-HEART is part of the 5G Infrastructure Public Private Partnership (5G PPP) ICT-19 (phase 3) projects [3], and its distinct objective is to deploy an extended set of innovative use cases pertaining to the Healthcare, Aquaculture, and Transport vertical industries and perform validation trials on real 5G pilot networks around Europe. This includes the complete life-cycle management, i.e., the requirement analysis, design, development, integration, testing, and trailing, of multiple concurrent and co-located vertical applications over the same network infrastructure. Hence, the outcome of the 5G-HEART project will build the foundation for the delivery of end-to-end 5G-enabled vertical services, capitalizing on a service-aware network infrastructure of elastic, software-driven, and programmable capabilities. Accordingly, the 5G-HEART project will contribute to the empowerment of the vertical industries under consideration, shedding light on similar approaches when targeting different use cases and verticals.

Our work focuses on the Transport vertical industry, which enabled by the 5G communications systems is expected to drive transformational changes and bring social, economic, and industrial benefits to the economies that will take the lead in adopting the latest virtualization and programmable technologies. More precisely, we scrutinize four major vehicular services, namely the “Platooning”, “Autonomous/Assisted Driving”, “Support for Remote Driving”, and “Vehicle Data Services”, each of which provides its own perspective and poses its specific requirements to the design of the overall system, ranging from the application development to the network infrastructure deployment. Our study initially focuses on mapping the qualitative user requirements to the quantitative network Key Performance Indicators (KPIs) for each of the aforementioned vehicular services. Subsequently, useful observations regarding the orchestration of the underlying physical network infrastructure are derived, especially emphasizing the initial network slice definition and dimensioning. A preliminary version of this work has been presented at the 2nd International Workshop on Real-life modelling in 5G networks and beyond (REFRESH 2021) [4].

In this paper, we go one step further compared to the aforementioned initial study. Capitalizing on the insights gained from this theoretical analysis, we experimentally explore the setup and configuration of the underlying network infrastructure that is intended to meet the diverse requirements of the aforementioned vehicular services. Additionally, preliminary validation trials and measurements on a real 5G facility complement our approach. Apart from these two striking differences, we further fine-tune and organize the requirement analysis methodology in a step-wise sequential pipeline that captures and represents the underlying procedure in a standard and easily reproducible way. Moreover, we expand on the necessary technical background and extend the analysis and interpretation of our findings. In this way, we are able to demonstrate the complete life-cycle of the development process followed within the context of the 5G-HEART project, starting from the design of the targeted services based on the user requirements and the identified network KPIs and concluding with the experimental validation and performance evaluation. Our main goal is to provide a holistic and systematic overview of the way in which 5G can enable the industry verticals and outline practical guidelines and best practices.

### 1.1. Related Work

In recent years, the advent of 5G has led to an increased interest in future applications that can fully utilize the extended capabilities it offers and has given rise to new challenges [5]. Specifically in the context of industrial communications, several studies have focused on describing novel use cases and scenarios involving vertical markets such as automotive, healthcare, manufacturing, food and agriculture, public transportation, media, and more [6,7,8,9]. For example, in [10], the authors present three novel Cooperated, Connected, and Automated Mobility (CCAM) use cases in a cross-border setting, running on top of the 5G communication infrastructures connecting three neighbouring cities. Apart from illustrative examples of the envisaged applications, a suitable set of metrics, target requirements, and KPIs has also been investigated, along with the enabling technologies needed for meeting these more stringent conditions [8,9,10,11,12]. As a representative example, an Artificial Intelligence (AI)-driven automated 5G end-to-end slicing solution for supporting multiple vertical services is introduced in [13]. The proposed AI-enabled closed-loop for service management is able to assure Service Level Agreement (SLA) compliance by incorporating features such as an automated Radio Access Network (RAN) orchestration and control and the multi-domain aggregation of services and resources from different providers.

Considerable research attention has been directed toward the architectural design of user applications and the technical aspects of network deployments, highlighting the importance of novel features of the 5G New Radio (NR) specifications [12,14,15,16]. Among the latter, the most critical ones are considered to be Multi-Access Edge Computing (MEC), which moves the computing resources closer to the user [17], and the Radio Access Network (RAN) slicing that enables the satisfaction of heterogeneous service requirements while sharing the same radio and processing resources [18]. Last but not least, a smaller number of studies has been devoted to the validation trials and field experiments related to the next generation wireless mobile telecommunications technologies under different vertical industries and applications: (i) remote control of Automated Guided Vehicles (AGVs) in factories [19]; (ii) Vehicle-to-Vehicle (V2V) direct communications for truck platooning [20]; and (iii) Massive MIMO in dense-urban, maritime, and high-mobility environments [21].

Particularly for the Transport vertical, the advantages and benefits of using 5G technology to enable Vehicle-to-Everything (V2X) services compared to existing alternatives, such as Fourth Generation (4G) cellular communications and non-cellular Dedicated Short-Range Communications (DSRC), have been extensively explored thus far [22,23,24]. In [25], the authors discuss the main use cases of 5G V2X and analyse their requirements, aiming to identify the gaps of the existing communication technologies. In this context, the 3rd Generation Partnership Project (3GPP) examines the network requirements that are imposed on the 5G infrastructure by a set of enhanced V2X scenarios [26]. Finally, in [27], the authors focus on the latency-critical V2X use cases. Compared to these “fragmented” studies, in this paper, we present a common methodology that, apart from the explicitly examined use cases, can be readily applied to other unexplored emerging scenarios, while at the same time we perform a comprehensive end-to-end analysis starting from the requirements gathering process and leading to validation trials with real pilots.

### 1.2. Contributions and Outline

In this paper, we focus on the design, development, and evaluation of four 5G-enabled use cases/service categories in the Transport vertical. In greater detail, we identify an extensive set of distinct underlying functionalities that are required to carry out each envisaged use case, referred to as scenarios in the following. These scenarios are characterized by different user requirements and KPIs, allowing us to map them into different 5G network slices and macroscopically better utilize the network infrastructure upon their final deployment. To achieve this, a comprehensive description of the considered scenarios for each use case is provided, along with the network KPIs identified via a 4-step methodology, enabling their final mapping to the generic 5G network slices. This thorough theoretical analysis primarily distinguishes our work from the existing literature and can provide useful insights to the industry that deals with either the user application or the network deployment side, promoting the synergy between the Telecom and Transport verticals.

Apart from the theoretical analysis, our work goes one step further and also accounts for the technical description and analysis of the network setup used to test and validate the performance of the use case scenarios. The purpose of the experimental part of the paper is not to present an optimized network configuration and related performance results for all the considered vehicular services. Instead, the experimental part tries to provide a practical reference point for the reader regarding the expected performance of early 5G network deployments operating with a similar configuration as presented for the test network. As the employed equipment is the same as the one found in the majority of the early public 5G deployments around the world, the provided best-case results are used as the upper bound of the performance that a commercial user can expect to achieve in a public network. Through the provided real-world performance reference, the conclusions made using the methodology introduced during the first half of the paper are validated and further discussed, while potential solutions from the 5G specification roadmap are provided to address the observed shortcomings and identified gaps with the goal of supporting the future large-scale deployment of the trialled vehicular services.

The main contributions of our work can be summarized as follows:

- We study four vehicular service categories of the Transport vertical, each one entailing a few scenarios where the focus is on a specific service or functionality. In particular, we perform a first-pass analysis of the desired functionality, the operational environment, and the user requirements of each scenario within each major vehicular service category (Section 3 and Section 4).
- We define and analyse the network Key Performance Indicators (KPIs) emerging in the considered scenarios of each vehicular service category. To that end, we devise a step-wise methodology for mapping the mostly high-level functional user requirements to more network-specific KPIs and we explore their interrelations (Section 2.3 and Section 4). The output of this effort is expected to be used for the evaluation of the forthcoming 5G-HEART trials.
- We pave the way towards network slice dimensioning. Namely, we determine and present the values of the network KPIs that will be required to be concurrently satisfied by the multiplexed virtualized and independent logical networks on the same physical network infrastructure, as allocated by the providers/operators to each specific scenario (Section 4 and Section 5).
- We discuss the impact of the derived network KPIs to the underlying physical infrastructure, providing insights for future enhancements to the employed architecture and the services currently foreseen in the 3GPP Release 15 and 16 (Section 5).
- We describe the configuration of a preliminary testing network setup, providing a fully controlled 5G network infrastructure based on commercial 5G equipment and spectrum (Section 6).
- Finally, we present the initial trials taking place within the context of the 5G-HEART project, aiming to evaluate the suitability of the preliminary 5G test configuration for supporting the examined vehicular services in order to guide the subsequent more advanced trials using optimized 5G networks (Section 7).

The remainder of this paper is structured as follows. Section 2 provides the technical background and methodology related to the network KPIs requirement analysis. The examined vehicular service categories are presented in detail in Section 3. Section 4 identifies the most stringent KPIs for the Transport vertical as a whole and explores how they can be fulfilled in the context of the generic 5G services. Section 5 highlights considerations regarding the overall infrastructure requirements and the envisioned network slices. The employed 5G network setup is detailed in Section 6, while the corresponding trials and performance evaluation are presented in Section 7. Finally, Section 8 concludes the paper identifying future research directions.

## 2. Technical Background and Methodology

In this section, we first present the list of target KPIs together with the three generic 5G service paradigms with a focus on their heterogeneous sets of requirements. Then, we present the systematic methodology we devised for the analysis of the requirements of the examined vehicular service categories and the subsequent initial network slicing design.

### 2.1. Quantitative Assessment via Key Performance Indicators

In the following, the definition and explanation of the considered network KPIs, which form the basis for the assessment of the satisfaction of the network requirements in 5G-HEART, are given in accordance with the latest standardization efforts [28,29] and 5G-PPP activities. Each of the network KPIs can be mapped to one or more high-level operational requirements from the perspective of the stakeholders and vice versa:

- *Throughput Downlink (DL)/Uplink (UL)* (Mbps): the number of correctly received bits over a certain period of time at the respective direction (application layer).
- *Latency* (ms): the time it takes for a transmitted data packet to reach its destination.
- *Reliability* (%): the success probability of transmitting a small data packet from the radio protocol layer 2/3 Service Data Unit (SDU) ingress point (e.g., from Internet Protocol (IP) to Service Data Adaptation Protocol (SDAP) for the 5G NR user plane) to the radio protocol layer 2/3 SDU egress point of the radio interface (e.g., from SDAP to IP for the 5G NR user plane) within a certain delay [26].
- *Mobility* (km/h): the maximum user speed at which a defined Quality of Service (QoS) can be achieved.
- *Location Accuracy* (m): the accuracy with which location information is provided to the end device/user.
- *Connection Density* (devices/km^2^): the total number of devices fulfilling a target QoS per unit area (per km^2^).
- *Interactivity* (transactions/s): the number of issued commands/requests and received acknowledgements per device, within a short period of time.
- *Area Traffic Capacity* (Mbps/m^2^): the total traffic throughput served per geographic area.
- *Security/privacy*: the protection of the usability and integrity of user data, equipment and network, as well as the privacy of user identity and information.

Table 1 presents the qualitative characterization (i.e., “Low”, “Medium”, and “High”) of the network requirements that is used and referred to in the remainder of the paper. Note that the considered peak value for connection density is based on the worst case US Freeway scenario that does not include arterial roads (i.e., on-ramps): 5 lanes in each direction or 10 lanes total per highway, for up to 3 highways intersecting = 3100 to 4300 vehicles per square kilometer [26].

### 2.2. 5G Generic Services

There are three 5G use-case driven sets of services considered as cornerstones that a 5G network aims to provide, namely [30]:

- *Enhanced Mobile Broadband (eMBB)*: This service aims at scenarios that are data-driven and require stable connections with high peak data rates across a wide coverage area, as well as moderate data rates for cell-edge users. Representative examples of such bandwidth-intensive services and applications include new immersive experiences such as Augmented Reality (AR) and Virtual Reality (VR) and access to resource-intensive multimedia content and data, such as Ultra High Definition (UHD) video sharing (e.g., 4K and 8K). The target KPI values are determined as follows: 100 Mbps for user experienced data rate with up to 20 Gbps in DL (peak) and 10 Gbps in UL (peak), 10 Mbits/s/m^2^ for area traffic capacity in DL, down to 4 ms for latency in both UL and DL, and up to 500 km/h for mobility.
- *Massive Machine Type Communications (mMTC)*: This service aims at scenarios characterized by a massive number of low-power devices in a small area, required to sporadically transmit a relatively low volume of delay-tolerant data. mMTC regards mainly applications in wearables and sensor networks. The main KPIs for the mMTC services include increased connection density, expanded coverage, and extended battery life. Taking into account the proliferation of IoT terminals, a target value for connection density of 1,000,000 devices/km^2^ (or equivalently 1 device/m^2^) is set for urban environments [29]. Regarding battery life, mMTC devices are required to operate for 10 to 15 years without changing or charging batteries. The coverage target of mMTC is defined in terms of 164 dB of Maximum Coupling Loss (MCL) (i.e., maximum total channel loss between the user equipment and base station antenna ports at which the data service can still be delivered). Finally, latency for the infrequent small packets shall be down to 10 ms in the UL.
- *Ultra-Reliable and Low Latency Communications (URLLC)*: This service aims at supporting low-latency transmissions with extremely high reliability. Indicative examples of applications with such requirements include the remote control of critical infrastructure, remote medical procedures, and transportation safety. In URLLC, the critical KPIs include latency and reliability with target values of 1 ms and 99.999%, respectively. To meet these stringent requirements, a set of enablers are required, including edge caching, computing and slicing, short Transmission Time Interval (TTI), mini-slots, and flexible numerology. Such capabilities, along with the increased synchronization and location accuracy provided by URLLC, can be utilized in high mobility usage scenarios to enhance transportation safety, where high data rates can be more or less important on a case-by-case basis.

### 2.3. Methodology

**Figure 1:**
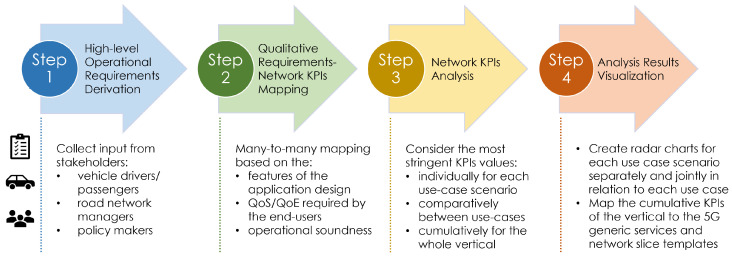
Overview of the proposed methodology.

As previously mentioned, one of the main objectives of this work is the specification and analysis of the requirements that the considered scenarios/use cases of the Transport vertical are putting on the network. To achieve this goal, the 4-step methodology described in Figure 1 is devised. Even though in this paper we examine four particular vehicular service categories/use cases of the Transport vertical, the proposed methodology can be readily applied to any kind of novel vertical use cases, enabling the systematic and thorough requirement analysis towards an efficient application design and network configuration of the common underlying physical infrastructure.

During the first step, the key involved stakeholders (e.g., vehicle drivers/passengers, manufacturers/technology partners, traffic/road operators, national/local authorities, and policymakers) determine a number of high-level operational requirements for each use case. These requirements mostly follow a qualitative approach focusing on the desired type and quantity of information that will be exchanged over the network and reflect user perception as well as traffic/safety regulations and operational soundness.

During the second step, the stakeholders’ qualitative indicators are mapped to a set of quantitative requirements for each considered scenario. The resulting network Key Performance Indicators (KPIs) are the ones that the underlying network infrastructure, the control/management planes, orchestration planes, and possibly slices will be called upon to fulfil. For mission-critical services, operational soundness is emphasized within the context outlined by the specific architecture and features of the employed solutions. The QoS/Quality of Experience (QoE) that a user perceives for the provided services [31] is taken into consideration in scenarios or components of scenarios where it is appropriate (e.g., streaming on-demand content, context-specific advertising, etc.). In the specific framework of 5G-HEART, the requirements of the considered innovative use cases are characterized based on the list of performance metrics presented in Section 2.1, which forms the basis for the assessment of the underlying network infrastructure.

The third step conducts an individual analysis for each use case, focusing on their striking differences in terms of network resource needs. This will be done while taking into consideration the three 5G use-case driven sets of services, their common set of capabilities and requirements [30], and associated values of characterizing KPIs [28,29]. Subsequently, the cumulative network requirements for the whole transport vertical are determined, providing a basis for the design of the required infrastructure building blocks and the overall 5G-HEART network architecture.

Finally, an educated comparison between the considered use cases is made based on a comprehensive visual representation. A set of multi-axes radar charts are produced for each scenario, then use case and ultimately the entire vertical, depicting the selected network KPIs and indicating the mapping to the 5G generic services and associated slice templates.

## 3. Next-Generation Vehicular Services

**Figure 2:**
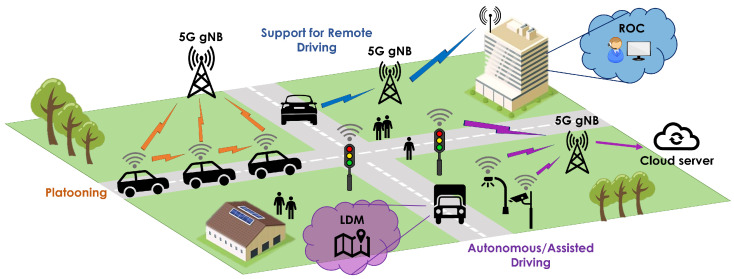
High-level overview of the advanced use cases expected to be supported by 5G V2X.

This section presents four major vehicular service categories that are expected to shape the frontier of the new transportation era thanks to the significant improvements that 5G networks bring in terms of connectivity and automation. Every service category comprises a set of use-case specific scenarios, each of them targeting a plethora of distinct functionalities. The considered use cases build on the enhanced V2X scenarios proposed by 3GPP in [26]. However, they are further analyzed in an extensive set of underlying scenarios and they are refined with real-world requirements and historical or even where possible real-time traffic data collected from the 5G-HEART consortium partners, which include owners and operators of roads and transport infrastructure covering urban, suburban, and remote areas. Subsequently, a comprehensive description of the use-case specific scenarios is provided, while the interested reader is referred to [32] for more details. Furthermore, Figure 2 provides a graphical illustration of some representative use-case scenarios that are advocated by the 5G-HEART efforts.

### 3.1. Platooning (T1)

Platooning refers to a group of vehicles (initially trucks, but later passenger cars as well) forming a tightly coordinated “train” with significantly reduced inter-vehicle distances. The platoon leader continuously shares status information (e.g., velocity, heading direction, and driving intention data) with the following vehicles, allowing for coordinated driving (i.e., vehicles can accelerate or brake simultaneously) with a much smaller headway, and thus alleviating the concerns regarding human reaction time and space. Benefits of platooning include energy savings and decreased fuel consumption due to the increased aerodynamic drag, reduced emissions, and improved road capacity and efficiency. Furthermore, by smartly managing the number of professional drivers needed for commercial vehicles, accident rates are reduced and productivity is enhanced. The specific scenarios considered in the context of platooning are presented below:

- *High bandwidth in-vehicle situational awareness and see-through for platooning* (T1S1 and T1S2): In order to provide enhanced situational awareness, collision warning, and see-through applications for platooning, the 5G-HEART project capitalizes on the potential of Augmented Reality (AR) by promoting the specific functionality of high-bandwidth in-vehicle real-time streaming. The real-world view is first captured and constructed by the leading platoon vehicle and then projected on the auditory and visual systems of the following platoon vehicles, making their passengers feel more secure and keeping their anxiety levels low. This can also improve safety via redundancy by extending object/event detection to the trailing vehicles, increasing at the same time the comfort of the passengers by enabling them to anticipate the maneuvers performed by the platoon leader in response to the changing operating conditions.
- *Dynamic channel management for traffic progression* (T1S3): This scenario devises a dynamic radio channel management approach for optimizing the assignment of radio channels to the Vehicle-to-Vehicle (V2V) and Vehicle-to-Infrastructure (V2I) communication links of vehicle platoons, aiming at the efficient utilization of the scarce radio resources, while at the same time accounting for the platoons’ mobility (since the platoons are moving, assigning fixed channels to them is not efficient). The considered centralized architecture consists of a V2X application that leverages indicative platoon status information (e.g., velocity, current location, and destination) to optimally assign in real time radio channels to the platoons, in a way that satisfies their needs for localized, low-latency, high-reliability and frequent communications. The product of this analysis is a Radio Environmental Map (REM) that merges geo-location information with the best radio channels and is continuously updated upon relevant changes.

### 3.2. Autonomous/Assisted Driving (T2)

The autonomous/assisted driving use case encompasses a wide variety of technologies and specific vehicular scenarios that share the end goal of enhancing the operation of connected and automated vehicles. This is achieved by the provisioning of services that support autonomous driving, such as network-assisted collision warning and avoidance systems. The support is based on the ample ambient information provided to the vehicles and their drivers from the surrounding environment and other road users in the area via the available 5G infrastructure. In more detail, the following scenarios have been considered by 5G-HEART:

- *Smart junctions and network assisted and cooperative collision avoidance (CoCA)* (T2S1 and T2S2): This scenario strives to prevent traffic accidents and assist cooperative automated driving functions as the vehicles traverse an intersection by providing them network-assisted safety information. This information includes precise digital maps of intersections, the status of traffic signals, and the locations of vehicles and Vulnerable Road Users (VRUs). Such safety information is time-critical and dense within a very short period of time and thus should be efficiently communicated to the vehicles, for instance, in the form of a Local Dynamic Map (LDM). Furthermore, smart junctions can contribute to improving and controlling the traffic flow by prioritizing certain public service vehicles (e.g., ambulances or fire trucks).
- *Quality of Service (QoS) for advanced driving* (T2S3): This scenario scrutinizes two key network functionalities to support advanced driving, namely (i) the initial negotiation of the connectivity and quality of service (QoS) levels offered by the network at the beginning of a given trip and (ii) the dynamic selection of the most suitable level of automation based on the predicted/anticipated conditions. Providing predictive alerts about connectivity and/or QoS degradation enables the timely change of automation level, ranging from fully autonomous to manual driving modes, with the goal of avoiding traffic hazards and collisions in the most effective way. This gives the human driver the ability to proactively take control of the vehicle and avoid the potential activation of pre-programmed emergency routines.
- *Human Tachograph* (T2S4): The scope of this scenario is to monitor the driver’s condition status based on wearable devices and to leverage this information to support safety applications. The driver’s alertness and fitness-to-drive are assessed by fusing data from wearable and on-board sensors that monitor physiological parameters (e.g., heart rate, body temperature, and skin conductivity) in real time. Beyond the current state of the driver, monitoring can be extended to include historical data, such as sleep quality, stress levels, and physical activity during the last couple of days, in order to identify potential risk factors. In the short term, such analysis can be exploited to prevent hazardous situations, warn about imminent dangers, and provide guidance and corrective actions. In the long term, the collected data can be used to devise strategies for preventing fatigue and speeding up recovery before and after driving.

### 3.3. Support for Remote Driving (T3)

Remote driving generally refers to the remote control of a vehicle using the available communication infrastructure by either a human operator or a cloud computing application. Contrary to fully autonomous driving, which requires sophisticated algorithms and complex subsystems to perform demanding real-time tasks (e.g., object identification and vehicle control), remote driving can be realized with much less complexity by including a human in the control loop. The human can easily recognize potential hazards, as long as he is provided with sufficient ambient information. Tele-operation can be offered as a stand-alone service to support various use-case scenarios, ranging from every-day automated transportation services to mission critical situations under harsh environmental conditions. More importantly though, the application of remote driving can serve as a complement or a back-up/on-demand service of the fully autonomous driving mode.

In this context, the development and delivery of *tele-operated support (T3S1)* constitutes an integral part of the 5G-HEART’s trialling plan. Real-time data feeds from the on-board sensors and High Definition (HD) cameras of the remotely-controlled vehicle, including the Global Navigation Satellite System (GNSS) position, are transmitted over the network to the Remote Operations Center (ROC). These data are eventually visualized and presented to a remote human operator, who sends commands to the vehicle in the opposite direction to control its speed and course and perform appropriate manoeuvrers. As a consequence, high bandwidth availability and increased achieved throughput are required for the uplink (UL), while a low latency requirement is imposed on both directions to enable the awareness, sense of presence, and spatial cognition of the remote operator (UL) and the real-time response of the vehicle to control commands (DL).

### 3.4. Vehicle Data Services (T4)

This service category includes a variety of distinct scenarios that aim at interconnecting potential third-party data sources, such as centralized online databases or distributed sensor networks, and the connected and automated vehicles via the available 5G infrastructure. The objective is to collect actionable information from all relevant parties (e.g., vehicles and road users) that can be used for enabling and facilitating advanced services pertaining to connected and automated vehicles. The specific scenarios and services envisioned by 5G-HEART are the following:

- *Vehicle prognostics* (T4S1): This scenario considers a road side unit (RSU) application that allows any passing vehicle to report its current operational state and receive a “Just in time repair notification” about any identified functional issue. To this end, the RSU application is connected to a local or a remote diagnosis/repair centre for the timely analysis of the reported data.
- *Over-The-Air (OTA) updates* (T4S2): This scenario provides Over-The-Air (OTA) software updates to vehicles, where the modules controlling the electronic functions, also known as Electronic Control Units (ECUs), are updated via 5G wireless connectivity in a transparent though secure manner. Since the vehicles do not need to be recalled by the manufacturer or be transferred to a service centre, these OTA updates are expected to significantly reduce maintenance costs.
- *Smart traffic corridors* (T4S3): This scenario focuses on the environmental benefits of collecting and analysing the vehicles’ historical and real-time data. More precisely, data collected by the vehicles can be used to intelligently route them with the goal of relieving congested areas and reducing emissions. Moreover, local administrators can leverage the extracted information to further optimize road maintenance.
- *Location-based advertising* (T4S4): This scenario studies the deployment of location-based servers that leverage the readily available vehicle and passenger information to stream on-demand content, context-specific advertising, and/or traffic guidance to geo-targeted groups. Such functionality becomes more important in the envisaged car-sharing models, where vehicles are temporarily rented and the routes they follow change frequently depending on the current passengers.
- *Vehicle-sourced High-Definition (HD) mapping* (T4S6): This scenario performs crowd-sourcing in collecting, maintaining and consolidating up-to-date data from the vehicles’ on-board sensors and cameras for the construction of accurate and dynamically configured HD maps. The constructed HD maps of roads and transportation infrastructure can be used to support the various autonomous driving-related services of the Transport vertical.
- *Environmental services* (T4S7): This scenario promotes the use of vehicles as a real-time source of rich weather and environmental sensor data that can be used for the construction of hyper-local weather maps. Such maps can be useful in aiding day-to-day driving and improving road maintenance processes.

## 4. Network Key Performance Indicators Analysis

**Figure 3:**
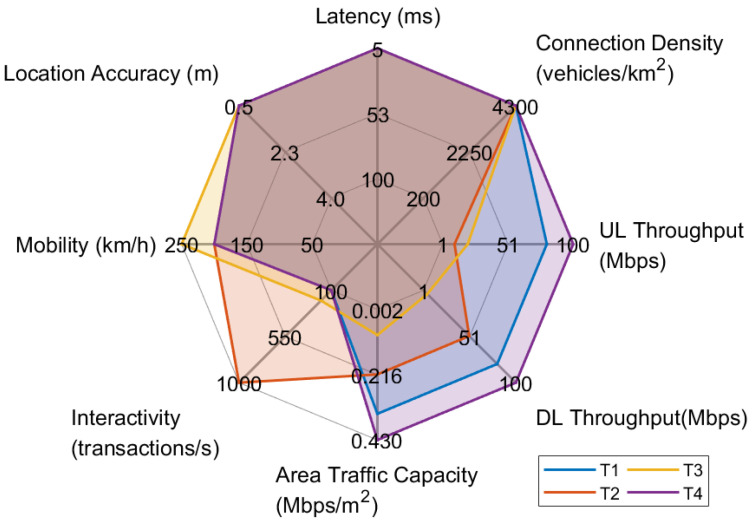
Radar chart of transport use cases.

The aforementioned vehicular services—or equivalent use cases—have different objectives, posing specific requirements to the overall system architecture. Accordingly, the qualitative requirements collected from the stakeholders participating in the 5G-HEART consortium are mapped to measurable network KPIs with specific target values, following the methodology presented in Section 2.3. The most stringent values of the selected KPIs for each use case/service category, calculated by overlapping the determined values of the corresponding individual scenarios, are presented in Table 2 and summarized in the form of a radar chart in Figure 3. In the following, we elaborate more on the explanation and interpretation of the resulting KPIs according to each use case’s main objective and functionality and based on the users’ expectations upon usage.

From the graphical representation of the network KPIs in Figure 3, it can be easily verified that the use cases T1 and T4 pose generally similar requirements and, specifically, yield the most stringent requirements in terms of the achieved UL/DL throughput. In greater detail, the Augmented Reality (AR) features along with the Vehicle-to-Vehicle (V2V) and Vehicle-to-Infrastructure (V2I) communications required for see-through and situational awareness in the use case T1 of platooning impose the need for increased UL/DL throughput up to 80 Mbps. Similarly increased UL/DL throughput values are found in use case T4, dictated primarily by the transmissions involved with the proposed vehicle sourced HD mapping in the uplink and the location-based advertising and over the air updates in the downlink direction. Regarding the use case T2, high DL throughput (up to 50 Mbps) is required by the Vehicle-to-Everything (V2X) application that reassures the sustainability of the QoS in the advanced driving scenario, while the instrumental and sensory data and video streams that are communicated to the ROC in the use case T3 of remote driving, justify the comparatively low required UL throughput (up to 20 Mbps).

Considering the remainder of the examined network KPIs, all vehicular use cases exhibit an increased need for medium latency (down to 5 ms), high location accuracy (reaching up to 0.5 m), and high mobility (200 km/h), in order to satisfy the user requirements. Additionally, the majority of the use cases require high reliability that can reach up to 99.99999% for the T1 and T2 specific use cases of platooning and advanced driving. This extremely high reliability requirement is paved by the desired real-time continuous operation, as well as the envisioned provision of personalized location-based services that are coupled with the high mobility of the vehicles. The peak connection density has been calculated following a similar procedure for all the use cases, considering the worst case of a five-lanes highway road for each travelling direction [26] and the most stringent area traffic capacity of 0.43 Mbps/m^2^ need, presented in the T4 use case. Subsequently, the peak connection density value is defined as equal to 4300 vehicles/km^2^ for all use cases.

Table 2 and Figure 3 also highlight the conservative requirements in terms of interactivity for all use cases except T2. The safety information exchanged between the vehicles and the network infrastructure, such as precise digital maps of intersections, the status of traffic signals, the locations of vehicles and vulnerable road users, and the drivers’ physiological status, is related to the smart junctions and network assisted and cooperative collision avoidance (CoCA) and the human tachograph scenarios and consists of payloads that are time-critical and dense within a very short period of time. As a consequence, the use case T2 is the only one with a demand of high interactivity (up to 1000 transactions/s). Finally, high security/privacy is needed across the whole vertical, as confidential/sensitive data is exchanged between the drivers/passengers and vehicles, intended for real-time operation.

## 5. Unified Physical Network Infrastructure

The 5G architecture fosters the growth of individual vertical sectors through provisioning services tailored to their needs and is currently under specification by the standard bodies. In view of tailoring the network deployment to provide customized vertical services, anticipating to provide stakeholders with new revenue streams, the generic 5G service types, and the overall system architecture (i.e., mobile edge/fog computing and network slicing) will be inevitably reconsidered. To this end, the resources allocated to different services have to be precisely defined, ensuring the desired QoS/QoE for each potential application, with the minimum possible resource consumption and at the lowest possible cost. Accordingly, in this section, we focus on a first assessment of the implementation needs of the several use cases comprising the unified physical network infrastructure, which leads to a preliminary dimensioning of the respective network slices. Each slice can be considered an end-to-end isolated logical network involving a specific collection of network functions and resource allocation modules [33], which is accommodated over the shared network infrastructure. Additionally, the selection of different enabling technologies needed to achieve the desired enhanced performance and flexibility is discussed.

**Figure 4:**
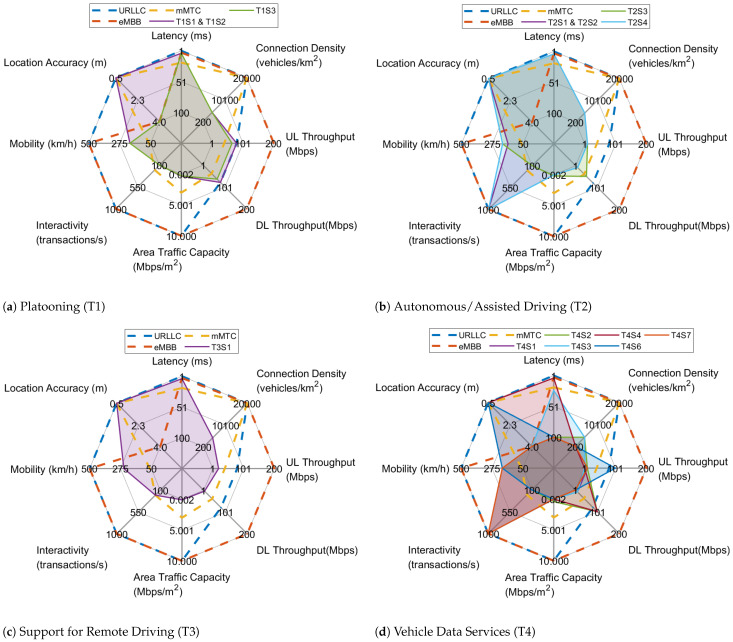
KPIs of Transport use cases and respective scenarios.

Figure 4 provides an overview of the target KPIs for each use case/vehicular service category and its component scenarios, together with the values achieved by the three 5G generic services. As can be seen, each scenario has its own focus and poses specific requirements suggesting the mapping to a generic service type/slice that is best suited to meet them. Different scenarios of the same use case can be mapped onto different 5G generic services, thus motivating the combination of suitable primitive slices for serving the entire use case. In the following, we attempt to perform a preliminary slice dimensioning by determining the appropriate combinations of 5G generic service types and associated primitive slices required for the concurrent support of the examined use cases and their component scenarios over the same physical network infrastructure.

**Figure 5:**
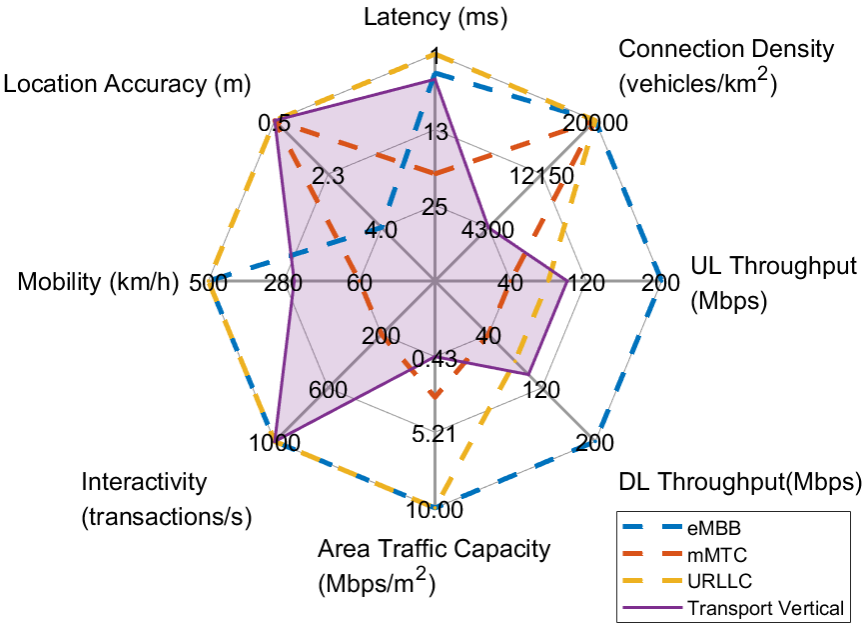
KPIs of the Transport vertical and related 5G service types.

Figure 5 presents the aggregated KPIs of the four individual vehicular services in relation to the three 5G service types. Each use case presents different requirements, demonstrating that a combination of eMBB, URLLC, and mMTC slices is required for the entire vertical, paving the need for subsequent network slices tailored to its particular KPIs. Specifically, URLLC and eMBB combined slices are needed in order to meet the target values of latency, mobility, UL/DL throughput, and reliability, while the need for mMTC slice is suggested by the demand for high location accuracy and the use of a potentially large number of multiple sensors.

The eMBB slice requires considerable bandwidth to support high-data-rate services, such as high-definition video streaming at varying mobility levels. As a result, a caching function together with data and cloud units are needed to assist control functions in implementing eMBB slicing services. On the other hand, URLLC slices serve applications that exhibit high sensitivity in terms of reliability, low latency, and security, such as autonomous driving, V2X communications, and Remotely Operated Vehicles (ROVs). Hence, to facilitate the needs of a URLLC slice, all dedicated functions can be instantiated at the network edge. Regarding the mMTC slice, which serves a large number of devices (e.g., sensors or wearables), a high level of connection density is required, with low demands in data rate and high energy/power efficiency.

Apart from this preliminary dimensioning of potential network slices required for servicing the different use cases and their component scenarios, several other recommendations derive from the KPIs’ analysis regarding the orchestration of the future network infrastructure towards meeting the diverse coexisting applications’ needs. Firstly, the stringent end-to-end latency requirements dictate the utilization of storage and computational resources in close proximity to the network edge and the end-users, migrating dynamically to the potential optimal locations. Secondly, seamless connectivity and service continuity with respect to the available communication, information, and computing resources should be pursued as a means of eliminating the detrimental effects of end-users’ high mobility in the experienced throughput and location accuracy. Last but not least, the coordinated deployment of antennas in terms of their number and types appears to be crucial not only for the resulting communications’ reliability but also for the achieved energy efficiency from the end-users’ perspective. The importance of the energy efficiency requirement is further stressed in mMTC environments, where multiple energy-constrained devices exist and operate. Thus, a balance between the contradicting interests of the network operators and the end-users should be targeted.

## 6. 5G Test Network Setup

**Figure 6:**
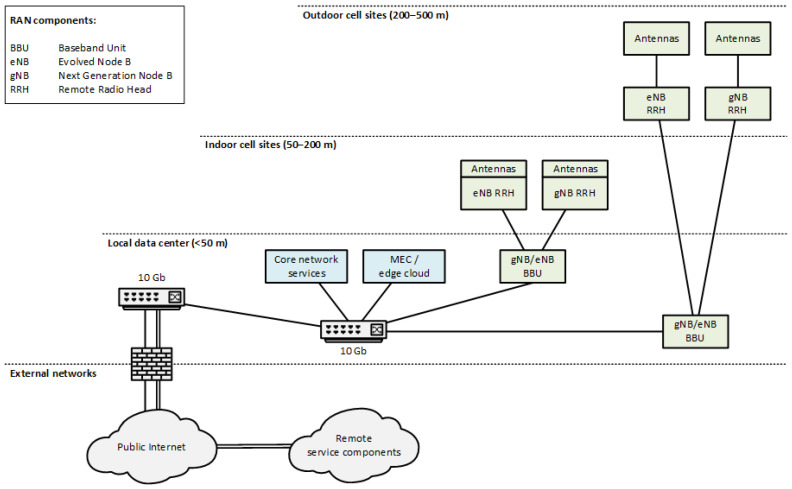
The 5G test facility architecture with estimated backhaul and fronthaul link distances.

The preliminary validation trials discussed in Section 7 have been performed at the 5G Test Network (5GTN) test facility in Oulu, Finland. The test facility architecture providing a fully controlled 5G network based on commercial 5G equipment and spectrum is presented in Figure 6. From the technological perspective, the infrastructure offers a realistic testing environment for a variety of vertical services that can be deployed into the architecture using the edge cloud resources provided by the facility or using a remote connection, e.g., by using Virtual Private Network (VPN) tunnelling over the public Internet. The 4G Evolved Packet Core (EPC) and 5G Core Network (5GC) Virtual Network Functions (VNFs) as well as the edge cloud environment are hosted as Virtual Machines (VMs) on dedicated server hardware in a local data centre residing in the same building with the test network cell sites. The virtualization of the services is based on OpenStack and VMware software platforms. The transport network infrastructure used for backhaul and fronthaul connections in the building is based on 10 Gb fibre links. The maximum link distances in the backhaul and fronthaul for the presented measurements are a few hundred meters. Measurement of the network traffic is performed at the IP-layer using synchronized software-based passive Qosium measurement probes [34], which can be activated in different locations of the overall data path at the edge cloud, 5GC, and RAN, as well as in the 5G User Equipment (UE). In the measurements reported in Section 7, one measurement probe was placed on the measurement server in the edge cloud and one on the UE residing in an otherwise empty 5G cell.

As the network is based on commercial equipment, a large variety of commercially available 5G UEs can be used to connect to it for testing purposes. The 5G RAN configuration used in the test facility at the time of the preliminary validation trials is presented in Table 3. Both the indoor and outdoor cell sites were utilized during the measurements and provided similar results. The 5G network was operating in Non-Standalone (NSA) mode, and the 4G anchor cell was operating at the 2.6 GHz frequency band (Band 7) with 10 MHz bandwidth.

The RAN configuration presented in Table 3 is tailored to provide an eMBB service which favours the Downlink (DL) direction over the Uplink (UL) direction in the allocation of transmission resources. All EPC and 5GC services, as well as the vehicular service components in the preliminary trials, are deployed in the edge cloud in order to minimize the end-to-end latencies experienced by the eMBB service users. The RAN part of the infrastructure does not include any optimizations specifically targeting low latencies or high reliability. The NSA network configuration providing a mobile broadband service represents the majority of the current commercial 5G deployments (at the time of this writing, only 4% of the network operators currently investing in 5G have actually launched a public 5G Standalone (SA) network [35]) and, instead of providing a fully optimized service for all the needs of the transport vertical, acts as an early performance reference for the discussed vehicle data services in this paper. However, considering the placement of all RAN, 5GC, and vehicular service components in the network edge, the preliminary measurement results presented in the following section must be considered the best-case performance for the presented 5G network configuration. In a large-scale commercial network infrastructure, the placement of the different architectural components must take into consideration the performance, scalability, and cost of the overall deployment.

The availability of the edge cloud environment is essential for the deployment of the vehicular services in use case T2 providing the required platform for local service data availability and, hence, low end-to-end service latency. For the deployment of the use cases T1 and T3, the service continuity and mobility management procedures must be optimized so that the required resources for high throughput services are always available during user mobility and recurring handovers. The vehicular services in use case T4 require optimization of the RAN transmission procedures to support either high spectral efficiency for high throughput data services or high energy efficiency for services based on data gathered from large quantities of energy-constrained sensor devices.

## 7. Preliminary Validation Trials and Measurements

The aim of the preliminary trials is to determine the best-case performance of the eMBB service provided by the 5G network in the utilized test facility and assess how well it is able to fulfil the requirements of the considered vehicular service categories, i.e., use cases T1–T4. By comparing the achieved performance against the target values presented in Table 2, we are able to identify the most significant performance gaps in the 5G technologies in their current early evolutionary state using an NSA network deployment and RAN configuration favouring downlink traffic. This configuration has been chosen for the preliminary baseline measurements as it has been traditionally used for offering mobile broadband services and is the prevailing configuration in the early commercial 5G network deployments. Based on the identified performance gaps and bottlenecks, we are able to further discuss the possible upgrades and reconfigurations needed in the network before it would be able to support the large-scale deployment of the trialled vehicular services. The focus in the presented discussion is on the means provided in the current or future 5G standards, as these are the options that are most likely available to the network operators during the coming years when their networks are first upgraded from 5G NSA to 5G SA and then from the current Release 16 functionality to Release 17 and beyond

As mentioned in the implementation requirement analysis of the transport use cases in Section 5, support for at least eMBB and URLLC slices is expected to be needed in order for the network to meet the diverse requirements related to DL/UL throughput, latency, and reliability as presented in Table 2. The measured average values for these essential network KPIs are shown in Table 4.

The best-case throughput results presented in Table 4 have been measured with a single user and full buffer test traffic in the DL and UL separately. The DL throughput achieved with the test network configuration is able to fulfil the requirements of all transport use cases considered in this study, but from the service scalability perspective, the amount of users that a single cell could support is still quite low. Based on the throughput targets presented in Table 2, the achieved cell capacity is enough to serve 5–11 users in the more demanding use cases T1, T2 and T4. However, the network configuration is potentially able to serve more than 100 users in use case T3. The achieved UL throughput is able to fulfil the requirements of the use cases T2 and T3, but even for these use cases, a single cell is only able to serve a few simultaneous users with the required service quality. Moreover, the UL capacity in the utilized test network configuration falls short of the more demanding requirements of the multimedia services utilized in the use cases T1 and T4.

Even though the maximum achievable data rates and cell capacity with the traditional broadband service configuration in a 5G NSA network deployment are not able to fully meet the requirements of the considered vehicular services, 5G standards still provide plenty of opportunities for reconfiguration and improvement. The utilization of larger bandwidth than the 60 MHz used during the measurements enables better throughput performance for the cell sites that have the spectrum resources at their disposal. For increased flexibility in the deployment of high bandwidth cell sites and more efficient utilization of the scarce radio spectrum, 5G standards provide enhanced mechanisms for Carrier Aggregation (CA), Dual Connectivity (DC), and Dynamic Spectrum Sharing (DSS), all of which can be used to ameliorate the achievable throughput in the considered use cases. The higher frequency bands have even larger bandwidths available than the sub-6 GHz low and mid bands, providing in this way additional deployment opportunities for densely populated and built areas. Additionally, enhanced Multiple-Input, Multiple-Output (MIMO), and Beamforming (BF) configurations in the air interface enable better throughput performance and robustness, especially in UL direction and high mobility scenarios.

The best-case latency results presented in Table 4 have been measured with a single user and continuous low data rate test traffic in the DL and UL separately. The average DL and UL latencies measured with the test facility setup are able to fulfil the 5 ms target listed for all use cases in Table 2. However, the achieved latency performance in the air interface is still heavily dependent on the cell load. When the full buffer test traffic utilized in the throughput tests is used in the cell, the excess delays related to the scheduling and queuing of the transmitted packets increase the average DL and UL latencies to 6.6 ms and 7.2 ms, respectively, which is already above the 5 ms required for the considered vehicular services.

Careful selection of the utilized scheduling algorithms and Quality of Service Class Identifiers (QCIs) for the different vehicular services can mitigate the noticeable negative impact of the cell load to the measured one-way latencies that are observed in the preliminary measurement results. Futhermore, in order to guarantee the required latency performance for the high priority services, the Admission Control (AC) mechanisms must be strict enough to avoid overloading at busy cell sites. When prioritizing URLLC over eMBB traffic during scheduling and configuring the frame structure for shorter Transmission Time Interval (TTI) than the one that was used in the test facility, the one-way latencies can be decreased. The Hybrid Automatic Repeat Request (HARQ) mechanisms can also be explicitly fine-tuned for low-latency applications to minimize the excess delay caused by possible retransmissions [36]. Further enhancements in the UL latency performance can be achieved with sub-slot-based or UL Configured-Grant (CG) transmissions [37].

When it comes to the reliability of the DL and UL transmissions, the achieved performance in the utilized test network configuration is still far from the required level for the considered transport use cases, especially when it comes to use cases T1 and T2. During the latency measurements, the reliability of 99.99% was achieved for 11 ms and 16 ms latency thresholds in DL and UL, respectively, but, overall, the communication reliability associated with the latencies below 10 ms seems to be the most difficult KPI to achieve with the first generation of 5G equipment. However, more sophisticated scheduling algorithms focusing on communication reliability will become available in the 5G equipment as the technology matures.

Concerning the ultra-reliable communications, the 5G standards provide several enhancements to MIMO, BF, joint transmission and interference mitigation schemes which can be used to increase the Signal to Interference and Noise Ratio (SINR) and the diversity gain at the receiver. In addition, reliability-specific enhancements are also available to link adaptation and HARQ mechanisms. Furthermore, several enhancements are available to make the control signalling more robust, as the requirement for high reliability and low latency in URLLC extends to the control plane [36].

Taking into consideration the measured performance of the 5G network in the preliminary trials and the mapping of the achieved performance figures against the target KPIs of the considered use cases, it is evident that the specific requirements of all the vehicular services cannot be simultaneously fulfilled with a single eMBB slice. The preliminary results support the conclusion made in Section 5 that at least eMBB and URLLC slices are needed for the network to be able to provide all the KPIs presented in Table 2 for the users of the vehicular services. Even though the architectural flexibility during network and slice deployment as well as the numerous configuration possibilities in the 5G RAN enable the fine-tuning of the network performance for the specific use case needs, throughput, latency, and reliability cannot all be optimized simultaneously. Consequently, a single network configuration is not appropriate, and, instead, network performance optimization needs to be performed by providing more than one logical network configurations to the users through slices. This way, the user will be able to choose one or more slices to serve the different use cases based on the KPIs they provide. In some of the use cases, the DL and UL service components can be allocated on top of different slice types to better meet the KPIs targets and optimize the end-to-end service quality.

Despite all the possibilities the 5G standards offer for different slice configurations in 5GC and RAN, specific attention needs to be paid to the placement and dimensioning of network functions and resources during slice deployment. Unlike in the test network utilized for the preliminary measurements in this paper, the network components in commercial network deployments are usually distributed on a larger geographical area, which results in additional delays in the fronthaul and backhaul portions of the infrastructure. In addition, the selection of the utilized transport network technology must take into consideration the strict delay requirements of the considered vehicular services. Based on the preliminary measurement results presented in Table 4 and the example transport latency values presented for a European mobile network deployment in [38], the 5 ms latency requirement defined for the vehicular services can only be met when both the user plane functionality and service data are available either directly at the cell site, as is the case for the test network introduced in Section 6, or at the tier 1 access site within 10 km of the cell site [39]. As the 5G technology evolves, the RAN latency can be pushed down to 1–2 ms with URLLC specific configurations. With a RAN configuration optimized for low latency communications, the user plane functions and vehicular service components could also be placed to tier 2 aggregation site, which typically resides within 40–100 km of the cell site. The possibility to host the service components at tier 2 instead of tier 1 requires approximately ten times fewer individually running service instances in the network, so depending on the size of the network infrastructure and scale of the provided vehicular service, the possibility to move them further away from the network edge can lead to significant savings for the operator in cost of offering the service to its customers [38]. However, these savings can only be realized if the operator’s transport infrastructure is able to support the traffic increase between the tiers.

## 8. Conclusions

New 5G mobile communications promise not only to augment existing applications but also to introduce a great number of novel services and use cases with strict requirements that previously could not be met. In this paper, we focused on the Transport vertical and the design, development, and evaluation of four envisioned vehicular service categories, examined within the context of the 5G-HEART project. More precisely, we devised a step-wise sequential methodology for quantifying the requirement analysis and mapping the high-level functional requirements to network-specific KPIs. Furthermore, we performed an initial network slice dimensioning based on the identified target values of the network KPIs that must be concurrently satisfied and the three 5G generic services/primitive slice templates (i.e., eMBB, mMTC, and URLLC). Finally, we provided considerations and guidelines regarding the common underlying infrastructure, while the findings of our theoretical analysis were experimentally verified by the preliminary validation trials realized in a real 5G test network.

To the best of our knowledge, this work is one of the first attempts to holistically investigate the impact of 5G on industrial communications and verticals, ranging from requirements gathering to network configuration and validation trials. The proposed approach is not only applicable to the Transport vertical but instead can be utilized to facilitate the evolution and implementation of various emerging use cases. Future research directions will focus on applying the devised methodology to the Aquaculture and Health verticals and further tailoring the network deployment and configuration towards a flexible solution that would efficiently accommodate customized 5G mobile network services for simultaneously supporting several vertical industries.

## Figures and Tables

**Table 1:** Qualitative characterization of network requirements.

Network Requirement	Ranges
DL throughput	Low ≤ 1 Mbps
	1 Mbps < Medium ≤ 10 Mbps
	10 Mbps < High
UL throughput	Low ≤ 1 Mbps
	1 Mbps < Medium ≤ 10 Mbps
	10 Mbps < High
Latency	Tight ≤ 5 ms
	5 ms < Medium ≤ 25 ms
	Loose > 25 ms
Reliability	Low ≤ 99.99
	99.99 < Medium ≤ 99.999
	99.999 < High ≤ 99.99999
Mobility	Low ≤ 50 km/h
	50 km/h < Medium ≤ 200 km/h
	200 km/h < High ≤ 500 km/h
Location accuracy	Low > 25 m
	1 m < Medium ≤ 25 m
	High ≤ 1 m
Connection density	No specific range; 4.3 × 10^3^ vehicles/km^2^ (peak)
Interactivity	Low ≤ 1 transactions/s
	1 < Medium ≤ 100 transactions/s
	100 < High ≤ 1000 transactions/s
Area traffic capacity	No specific range; 10 Mbps/m^2^ (peak)
Security/privacy	Low: Public
	Medium: Restricted
	High: Confidential

**Table 2:** 5G-HEART Vehicular Services’ Network Key Performance Indicators.

Network KPI	Units	Platooning (T1)	Autonomous/Assisted Driving (T2)	Support for Remote Driving (T3)	Vehicle Data Services (T4)
DL throughput	Mbps	80	50	5	100
UL throughput	Mbps	80	10	20	100
Latency	ms	5	5	5	5
Reliability	%	99.99999	99.99999	99.999	99.999
Mobility	km/h	200	200	250	200
Location accuracy	m	0.5	0.5	0.5	0.5
Connection density	vehicles/km^2^	4300	4300	4300	4300
Interactivity	transactions/s	100	1000	200	100
Area traffic capacity	Mbps/m^2^	0.344	0.215	0.086	0.43
Security/privacy	Public/Restricted/Confidential	Confidential	Confidential	Confidential	Confidential

**Table 3:** 5G test facility RAN configuration.

Network Parameter	Configuration Value
Frequency band	3.5 GHz (Band n78)
Channel bandwidth	60 MHz
Duplex mode	TDD
DL/UL ratio	7/3
Subcarrier spacing	30 kHz
Transmission time interval	0.5 ms
Modulation	256 QAM in DL
	64 QAM in UL
MIMO	4 × 4 in DL
	1 × 2 in UL

**Table 4:** Measured performance of a Rel-15 5G test facility.

Network KPI	Measurement Value
Best-case DL throughput	568 Mbps
Best-case UL throughput	63 Mbps
Average DL latency	4.0 ms
Average UL latency	4.7 ms
DL reliability	99.99% @ 11 ms
UL reliability	99.99% @ 16 ms

## Data Availability

Not applicable.

## Abbreviations

The following abbreviations are used in this manuscript:

5G	Fifth Generation
QoS	Quality of Service
QoE	Quality of Experience
5G-HEART	5G HEalth, AquacultuRe and Transport
5G PPP	5G Infrastructure Public Private Partnership
KPI	Key Performance Indicator
eMBB	enhanced Mobile Broadband
mMTC	massive Machine Type Communications
URLLC	Ultra-Reliable Low Latency Communications
CCAM	Cooperated, Connected and Automated Mobility
RAN	Radio Access Network
AGV	Automated Guided Vehicle
V2V	Vehicle-to-Vehicle
V2X	Vehicle-to-Everything
V2I	Vehicle-to-Infrastructure
V2N	Vehicle-to-Network
4G	Fourth Generation
DSRC	Dedicated Short-Range Communications
3GPP	3rd Generation Partnership Project
DL	Downlink
UL	Uplink
AR	Augmented Reality
VR	Virtual Reality
UHD	Ultra High Definition
REM	Radio Environmental Map
ROC	Remote Operations Center
HD	High Definition
GNSS	Global Navigation Satellite System
OTA	Over-the-Air
ECU	Engine Control Unit
CoCA	Cooperative Collision Avoidance
ROV	Remotely Operated Vehicle
MEC	Multi-Access Edge Computing
AI	Artificial Intelligence
SLA	Service Level Agreement
MCL	Maximum Coupling Loss
TTI	Transmission Time Interval
ADAS	Advanced Driver-Assistance System
CACC	Cooperative Adaptive Cruise Control
VRU	Vulnerable Road User
LoA	Level of Automation
RSU	Road Side Unit
5GTN	5G Test Network
VPN	Virtual Private Network
VNF	Virtualized Network Function
UE	User Equipment
NSA	Non-Standalone
SA	Standalone
5GC	5G Core
CA	Carrier Aggregation
DC	Dual Connectivity
DSS	Dynamic Spectrum Sharing
MIMO	Multiple-Input Multiple-Output
BF	Beamforming
QCI	Quality of Service Class Identifier
AC	Admission Control
HARQ	Hybrid Automatic Repeat Request
CG	Configured-Grant
SINR	Signal to Interference and Noise Ratio
